# P-1963. Changes in COVID-19 Sentiment and Behavior among Healthcare Professionals Over Time in a Rural State

**DOI:** 10.1093/ofid/ofae631.2122

**Published:** 2025-01-29

**Authors:** Brianna S Wright, Daniel Kang, Aaron Scherer, Melissa Ward, Daniel Diekema, Loreen Herwaldt

**Affiliations:** University of Iowa Health Care, Coralville, Iowa; University of Iowa, Bloomington, Illinois; University of Iowa, Bloomington, Illinois; University of Iowa, Bloomington, Illinois; MaineHealth; University of Iowa, Bloomington, Illinois

## Abstract

**Background:**

We assessed attitudes towards COVID-19 and use of COVID-19 infection prevention practices during the pandemic among employees at an academic health center in a rural state.

**Figure 1:**
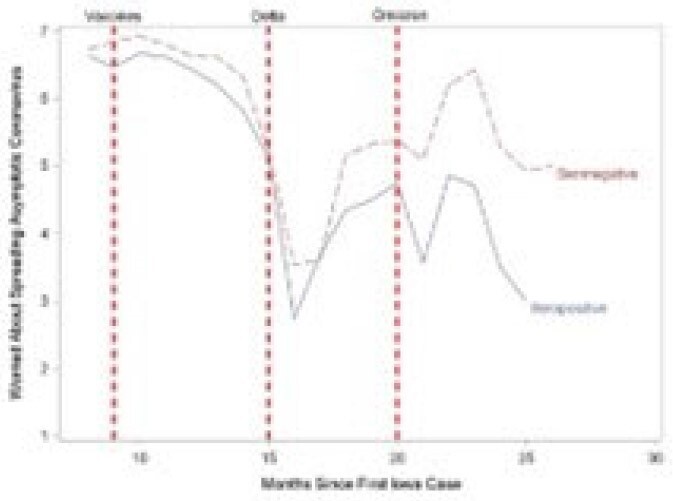
Reported Level of Worry about Spreading SARS- CoV-2

**Methods:**

We invited all employees aged >18 years reporting prior COVID-19 on a screening survey to participate and randomly selected other participants (Ps) from among those not reporting prior COVID-19 to create a study cohort of 302 participants. Ps completed surveys at 3-month intervals (T0, T3, T6, T9). We used the Chi-squared test to assess changes in Ps’ responses between individual time points.

**Figure 2:**
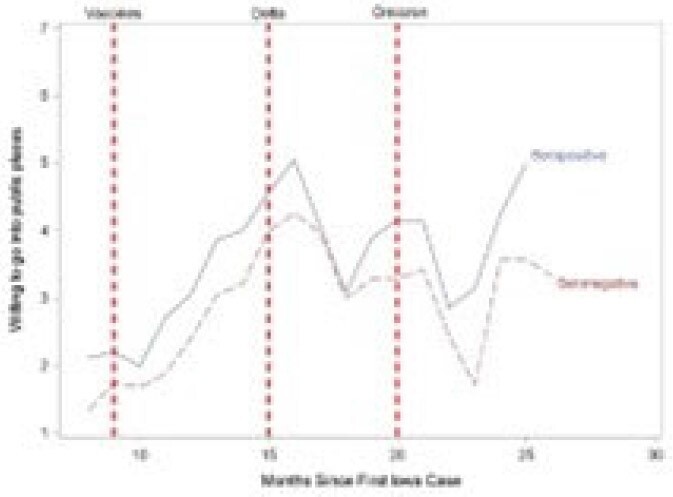
Reported Willingness to Go into Public Places

**Results:**

At T0 most Ps thought their risk of acquiring COVID-19 either in the hospital or the community was low (< 3%) and < 20% worried that they or their household members would “catch COVID-19.” However, 45% of Ps were worried about spreading SARS-CoV-2 asymptomatically.

Ps’ perceived likelihood of getting COVID-19 at the hospital and in the community did not change. The percent of Ps who were very worried about getting COVID-19 was significantly lower at T1 (1.7%), T2 (2.6%), and T3 (5.7%) than at T0 (11.3%; P < .05 for all). The percent of Ps who were very worried that household members would get COVID-19 decreased from T0 (16.6%) to T1 (8.0%; P = .03).

After vaccines were introduced, Ps tended to report being less worried about spreading COVID-19, more willing to go to public places and less likely to wear face masks in the public. Shortly after the Delta variant emerged, these sentiments changed direction but did not return to their baseline cautiousness level. Shortly before the Omicron variant emerged, the sentiments of those who were seropositive at any time and those who remained seronegative during the study period diverged, with the latter being more worried about asymptomatic spread, less willing to go to public places and more willing to mask in the community than those who seroconverted. Consistent with these changes, 21 of 26 (80.8%) seroconversions occurred during the Omicron surge.

**Figure 3:**
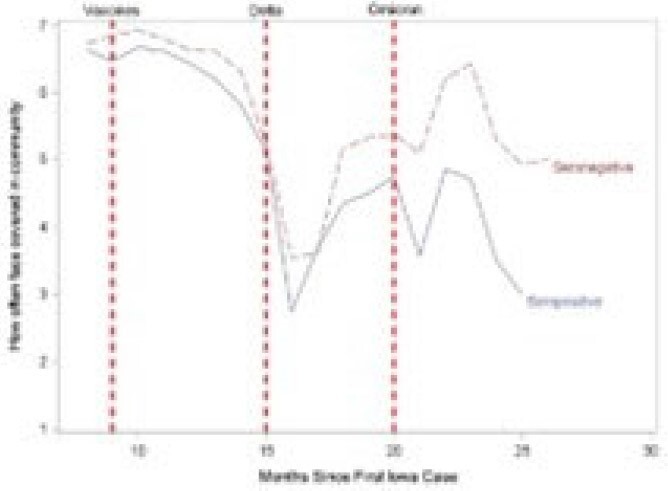
Changes in Reported Use of Face Coverings in Public

**Conclusion:**

Some attitudes and behaviors changed over time possibly related to vaccine availability and the predominant SARS-Co-V2 variant. Ps who remained seronegative tended to be more concerned about getting COVID-19 and were more likely to continue using COVID-19 infection prevention practices.

**Disclosures:**

Daniel Diekema, MD, MS, D(ABMM), Affinity Biosensors: Grant/Research Support|bioMerieux, Inc: Grant/Research Support Loreen Herwaldt, MD, 3M: Grant/Research Support|PDI: Grant/Research Support

